# Chronic HIV Infection Enhances the Responsiveness of Antigen Presenting Cells to Commensal *Lactobacillus*

**DOI:** 10.1371/journal.pone.0072789

**Published:** 2013-08-30

**Authors:** Lauren H. Nagy, Irina Grishina, Monica Macal, Lauren A. Hirao, William K. Hu, Sumathi Sankaran-Walters, Christopher A. Gaulke, Richard Pollard, Jennifer Brown, Maria Suni, Andreas J. Baumler, Smita Ghanekar, Maria L. Marco, Satya Dandekar

**Affiliations:** 1 Department of Medical Microbiology and Immunology, University of California Davis, Davis, California, United States of America; 2 Department of Internal Medicine, University of California Davis, Davis, California, United States of America; 3 Food Science and Technology University of California Davis, Davis, California, USA; 4 Becton Dickinson Biosciences, San Jose, California, United States of America; Emory University School of Medicine, United States of America

## Abstract

Chronic immune activation despite long-term therapy poses an obstacle to immune recovery in HIV infection. The role of antigen presenting cells (APCs) in chronic immune activation during HIV infection remains to be fully determined. APCs, the frontline of immune defense against pathogens, are capable of distinguishing between pathogens and non-pathogenic, commensal bacteria. We hypothesized that HIV infection induces dysfunction in APC immune recognition and response to some commensal bacteria and that this may promote chronic immune activation. Therefore we examined APC inflammatory cytokine responses to commensal lactobacilli. We found that APCs from HIV-infected patients produced an enhanced inflammatory response to *Lactobacillus plantarum* WCFS1 as compared to APCs from healthy, HIV-negative controls. Increased APC expression of TLR2 and CD36, signaling through p38-MAPK, and decreased expression of MAP kinase phosphatase-1 (MKP-1) in HIV infection was associated with this heightened immune response. Our findings suggest that chronic HIV infection enhances the responsiveness of APCs to commensal lactobacilli, a mechanism that may partly contribute to chronic immune activation.

## Introduction

Chronic immune activation is a major driver of HIV disease pathogenesis. It contributes to the persistence of viral reservoirs, incomplete immune restoration, as well as medical co-morbidities. Hallmarks of chronic immune activation, including increased immune cell activation and inflammation, can be seen in a wide spectrum of HIV-infected patients, including therapy-naïve patients and those who achieve complete viral suppression on long-term highly active antiretroviral therapy (HAART) [1]–[3]. Mechanisms contributing to chronic immune activation are multifactorial and include residual viral replication in tissues, reactivation of latent viral infections, and microbial translocation [4]–[6]. To date, the majority of investigations pertaining to chronic immune activation in HIV infection have focused on the role of CD4+ T-cells. In contrast, the role of antigen presenting cells (APCs) in chronic immune activation is not fully defined. A better understanding of functional impairment in APC immune recognition and response will provide insights into HIV pathogenesis and may identify novel therapeutic targets.

APCs, including myeloid dendritic cells (mDCs), monocytes, and macrophages, recognize pathogen-associated molecular patterns (PAMPs) through pattern recognition receptors (PRRs), including toll-like receptors (TLRs) [7]. Following pathogen recognition by PRRs, APCs generate a rapid immune response via signaling pathways, including NFkB and mitogen-activated protein kinase (MAPK), through phosphorylation of transcription factors that induce the production of inflammatory cytokines including IL-6, IL-12/IL-23p40, and TNFα [8]. This inflammatory response to pathogens leads to the recruitment of both innate and adaptive immune cells to the site of infection so that pathogen clearance can occur.

APCs have an important role in the host’s ability to distinguish between self versus non-self as well as pathogenic versus commensal bacteria. This function is essential for generating appropriate immune responses to pathogens while avoiding immune diversion to innocuous microbial stimuli and preventing chronic inflammation. The ability to distinguish between pathogenic and commensal bacteria is particularly important in the human gastrointestinal (GI) tract because it harbors over 10^14^ microorganisms [9]. Immune cells, particularly dendritic cells, in the GI tract influence the interactions between host and commensal bacteria enabling a symbiotic relationship critical for immune development and prevention of chronic inflammation and tissue damage [10], [11].

To date, the effects of HIV infection on the innate immune system and its response to commensal bacteria, including lactobacilli, are not fully defined. It is of particular importance to determine whether HIV alters APC functional responses to commensal bacteria because APCs may encounter these microorganisms or their microbial products in circulation or at the mucosal sites of infection [12], [13]. In addition, commensal *Lactobacillus* strains are consumed through diet as well as employed as probiotics, many of which are currently under evaluation in HIV clinical trials [14], [15]. Therefore, we examined the effects of HIV infection on the APC responses to different lactobacilli, including *Lactobacillus plantaru*m WCFS1, *Lactobacillus gasseri* 1SL4, and *Lactobacillus casei* BL23. Our findings identify heightened APC response to commensal *Lactobacillus* as a potential mechanism of chronic immune activation and inflammation in HIV infection.

## Results

### Increased Immune Cell Activation in HIV-infected Patient Groups

Peripheral blood samples were obtained from HIV-negative controls (HIV−, n = 40), HIV-infected therapy-naïve patients (n = 34), and HIV-infected patients receiving long-term HAART (n = 63). Patient groups and characteristics are presented in Table 1. HIV-infected patients, both therapy-naïve and HAART-treated, demonstrated varying degrees of peripheral blood CD4+ T-cell loss. Compared to HIV-negative controls, both HAART-treated and therapy-naïve patients demonstrated significantly decreased frequencies of CD4+ T-cells and increased CD8+ T-cell frequencies. While most of the HIV-infected, therapy-naïve patients had detectable plasma viral loads and low CD4+ T-cells numbers, four of the 34 patients had undetectable plasma viral loads and CD4+ T-cells counts >700 cells/mm^3^ despite being HIV-positive for over ten years. HAART-treated HIV-infected patients also showed a range of CD4+ T-cells counts, indicating variable levels of immune recovery. Significant increases in frequencies of activated CD4+ and CD8+ T-cells were also seen in HIV-infected, therapy-naïve patients. Higher frequencies of activated CD4+ T-cells were also detected in HAART-treated patients, reflecting the persistence of immune activation despite long-term therapy. Since a majority of the HIV-infected patients (both therapy-naïve and HAART-treated) had lower CD4+ T-cells frequencies and higher immune activation than HIV-negative controls, we present the APC functional data from HIV-infected patients collectively as an HIV-infected group.

**Table 1:** Patient cohorts and characteristics.

		Age	Males:Females	CD4+ T cell count cells/mm^3^	Viral load(HIV RNA copies/mL plasma)	%CD3+CD4+	%CD3+CD8+	%CD4+HLA-DR+	%CD8+HLA-DR+	Years diagnosed	Years on HAART	HAARTregimen
HIV-negative(HIV-)	n = 40	38(21–63)	17∶20	N/A	N/A	67.3%(46.7–91.9)	23.2%(4.4–46.0)	4.90%(1.44–22.0)	6.96%(0.55–28.50)	N/A	N/A	N/A
HIV-infected,therapy-naïve	n = 34	41(20–77)	28∶6	506(18–1353)	76,390(<50–976,464)	41.8%****(10.5–70.7)	44.0%****(17.2–66.4)	12.27%****(1.720–44.20)	20.20%****(1.24–58.70)	6.06(1–23)	N/A	N/A
HIV-infected,HAART	n = 63	50(30–64)	47∶13	497(96–1578)	<50	47.9%****(16.9–90.4)	40.0%****(3.4–74.4)	9.21%**(1.22–27.40)	9.69%†(0.12–32.20)	6.06(1–23)	6.96(2–20)	PI, NNRTI, combination

Age, T cell counts, viral loads, years diagnosed and years on HAART are expressed as mean values with the range indicated in parentheses. CD4+ T cell counts and plasma viral load are not applicable (N/A) for HIV-negative controls. Plasma viral load was under 50 HIV RNA copies/mL plasma in HIV-infected, HAART-treated group. (PI = protease inhibitor, NNRTI = non-nucleoside reverse transcriptase inhibitor, combination = PI+NNRTI). P values determined using ANOVA followed by Bonferroni’s Multiple Comparisons test (**P<0.01, ****P<0.001 compared to HIV-negative controls.

† P<0.001 compared to HIV, therapy naïve.).

**Figure 1:**
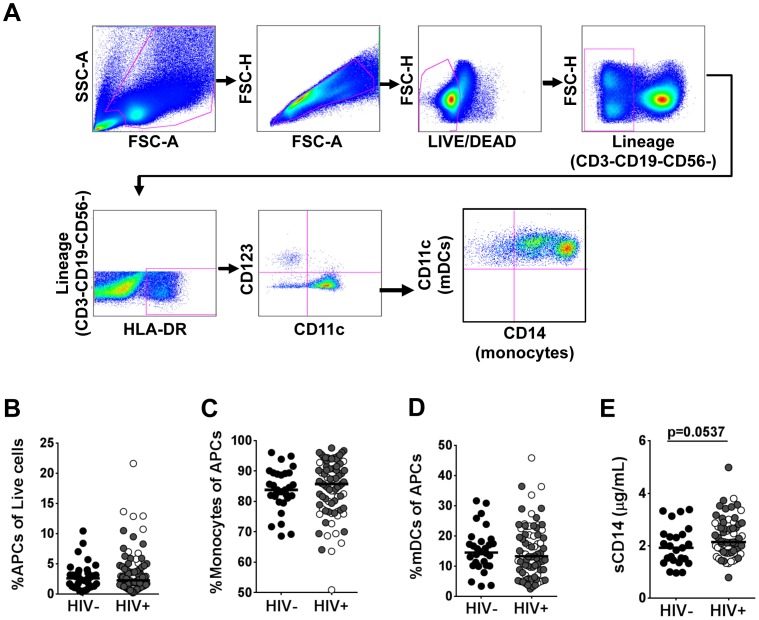
Distribution of antigen presenting cells in peripheral blood during chronic HIV infection. (**A**) Multicolor flow cytometric gating strategy for the identification of APC populations in peripheral blood. APCs (CD3-CD19-CD56-HLADR+CD11c+CD123-), monocytes (CD3-CD19-CD56-HLADR+CD123-CD11c+CD14+), and mDCs (CD3-CD19-CD56-HLADR+CD123-CD11c+CD14+) were detected following the exclusion of dead cells and CD3+, CD19+ and CD56+ cells. The HLA-DR-positive cells were gated for CD11c and CD123. Data were analyzed using FlowJo. (**B**) Percentages of circulating APCs (HIV− n = 37, HIV+ n = 94). (**C**) Monocyte percentage of the peripheral blood APC population. (**D**) Myeloid dendritic cell population of the peripheral blood APC population. (**E**) Plasma sCD14 concentrations as determined by ELISA (HIV− n = 25; HIV+ n = 66). Each dot represents an individual patient. In the HIV+ group, open circles represent therapy-naïve patients and closed circles represent patients on HAART. Bars indicate median value. P values determined using Mann Whitney U test. P values as indicated.

We focused our investigations on the effects of HIV infection on the APC population (CD3-CD19-CD56-HLADR+CD11c+CD123-). The multicolor flow cytometric gating strategy used to detect the APC population is included in Figure 1A. PBMCs were initially gated based on forward and side scatter and doublets were excluded to ensure that the analysis was based on single cells. Viable lineage-negative (CD3-CD19-CD56-) cells were assessed for expression of HLA-DR. From this population, APCs were defined as CD11+ and CD123-. No significant changes were found in the peripheral blood frequencies of APCs between the HIV-infected patients and the HIV-negative controls (Figure 1B). Further analysis showed that the APC population included both monocytes and mDCs. Monocytes, which were gated based on CD14 expression and included cells expressing both high and low levels of CD14, comprised a majority of the APC population (Figure 1C). Cells negative for CD14 but positive for CD11c were considered mDCs and comprised the remainder of the APC population (Figure 1D). Flow cytometric analysis showed no significant changes in the distribution of monocyte or mDC percentages in the APC population in HIV-infected patients compared to HIV-negative controls (Figure 1 C–D). In support of previous studies that have reported increased levels of innate immune cell activation [6], [16], [17], we found higher levels of monocyte activation marker, soluble CD14 (sCD14), in plasma samples of HIV-infected patients compared to HIV-negative controls (Figure 1E).

### Enhanced APC Inflammatory Response to Commensal *L. plantarum* WCFS1 in HIV Infection

**Figure 2:**
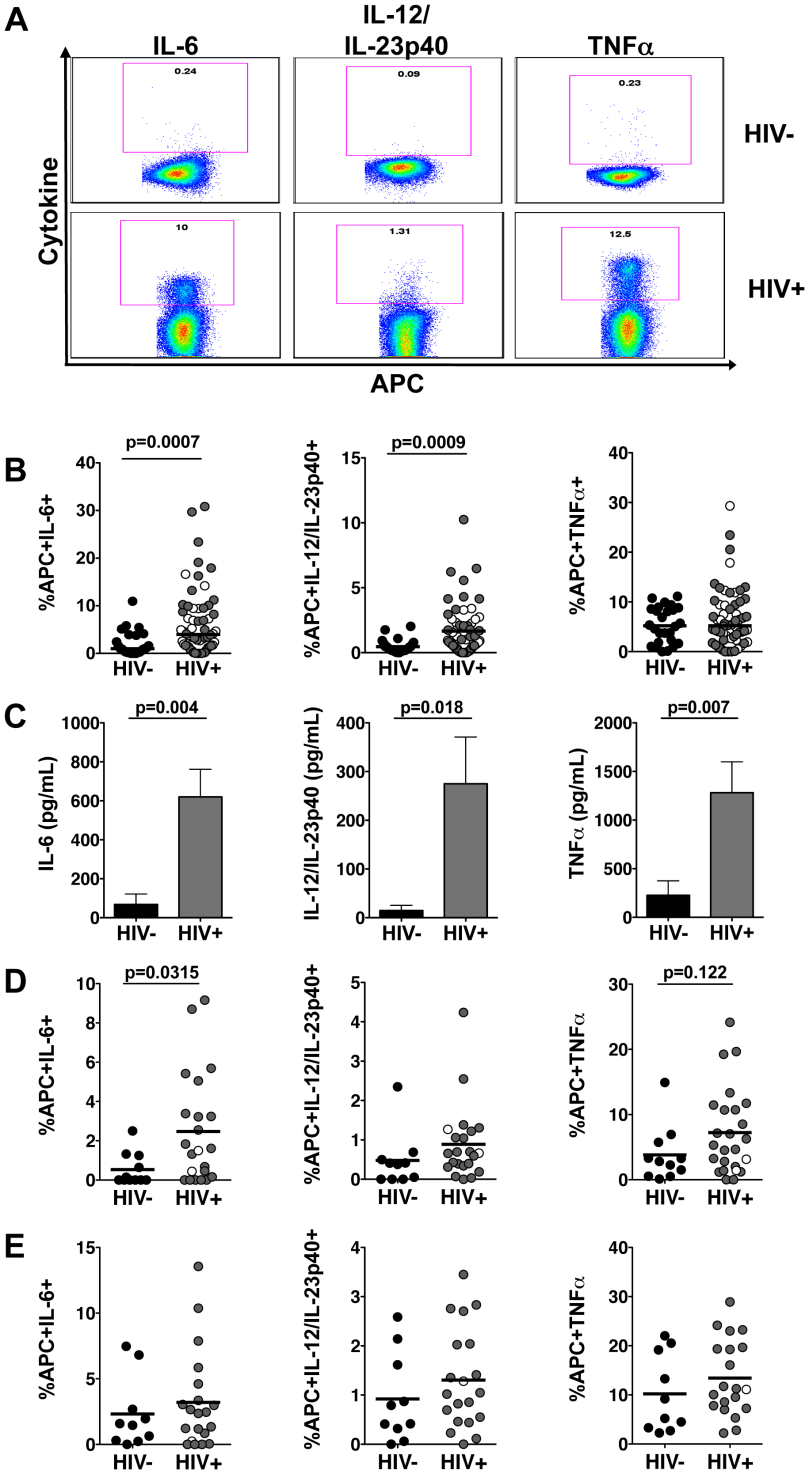
Enhanced inflammatory response by APCs from HIV-infected patients to commensal lactobacilli. (**A**) Representative flow cytometry plots of APCs producing proinflammatory cytokines in response to *L. plantarum* WCFS1. (**B**) Frequencies of APCs from HIV-negative controls (n = 29) and HIV-infected patients (n = 62) producing IL-6, IL-12/IL-23p40, and TNFα in response to *L. plantarum* WCFS1 determined by multicolor flow cytometry. (**C**) Concentrations of IL-6, IL-12/IL-23p40, and TNFα following stimulation with *L. plantarum* WCFS1 as determined by ELISA (HIV− n = 4; HIV+ n = 10). Frequencies of APCs from HIV-negative controls (n = 14) and HIV-infected patients (n = 23) producing IL-6, IL-12/IL-23p40, and TNFα in response to (**D**) *L. gasseri* 1SL4 and (**E**) *L. casei* BL23 as measured by multicolor flow cytometry. Each dot represents an individual subject. In the HIV+ group, open circles represent therapy-naïve patients, closed circles represent patients on HAART. Bars indicate median value. Bar graphs represent mean +/− SEM. P values determined using Mann Whitney U test, P values as indicated.

It is unknown whether HIV alters APC immune sensing and response to commensal lactobacilli. Therefore we examined APC inflammatory responses to several *Lactobacillus* species. Previous studies reported that stimulation with *L. plantarum* WCFS1 induces minimal inflammatory responses in PBMCs from healthy individuals [18]. Following exposure to *L. plantarum* WCFS1 [19], APCs from HIV-negative controls produced minimal inflammatory cytokine response, confirming that APCs in healthy individuals do not respond strongly to this *Lactobacillus* strain (Figure 2A). Remarkably, significantly higher frequencies of APCs from HIV-infected patients produced IL-6 and IL-12/IL-23p40 in response to *L. plantarum* WCFS1 as detected by multicolor flow cytometry (Figure 2B). In addition, analysis by ELISA revealed that PBMCs from HIV-infected patients released significantly higher concentrations of IL-6 and IL-12/IL-23p40 following stimulation with *L. plantarum* WCFS1 (Figure 2C). While no significant differences were found in the frequencies of PBMCs producing TNFα in response to *L. plantarum* WCFS1, cells from HIV-infected patients released significantly higher concentrations of TNFα (Figure 2B and C).

Several *Lactobacillus* strains have been shown to stimulate either pro- or anti- inflammatory responses in host cells [20], [21]. To assess any aberrant proinflammatory responses due to HIV infection, we focused on *Lactobacillus* strains that have been shown to induce anti-inflammatory effects. Therefore, we expanded the analysis of APC responses to *L. gasseri* 1SL4 (Figure 2D) and *L. casei* BL23 (Figure 2E). Mucosal administration of both *L. gasseri* and *L. casei* has been reported to reduce inflammatory responses such as oxidative stress, IL-17-driven inflammation, and other inflammatory infiltrates in inflammatory models of allergen-induced airway inflammation and DSS-induced colitis [22]–[24]. APCs from HIV-infected patients also produced an enhanced inflammatory cytokine response to *L. gasseri* 1SL4 (Figure 2D) and *L. casei* BL23 (Figure 2E) indicating that the enhanced inflammatory response by APCs from HIV-infected patients was not confined to *L. plantarum* WCFS1.

Analysis of the inflammatory response to lactobacilli by APCs was expanded to the monocyte and mDC populations (Figure S1). These data show that higher frequencies of monocytes produced inflammatory cytokines in response to the lactobacilli compared to the mDC population (Figure S1A and S1B). In addition, the data demonstrate higher frequencies of monocytes and mDCs from HIV-infected patients produced inflammatory cytokines in response to the lactobacilli compared to those from HIV-negative controls (Figure S1A and S1B). While the sample size was smaller, similar increased inflammatory responses were also observed in HIV-infected patients in response to *L. rhamnosus* GG (Figure S2A) and *L. reuteri* F275 (Figure S2B). Together, these findings suggest that HIV infection induces a heightened APC inflammatory response to commensal lactobacilli.

### Increased APC Expression of TLR2 and CD36 in HIV Infection

**Figure 3:**
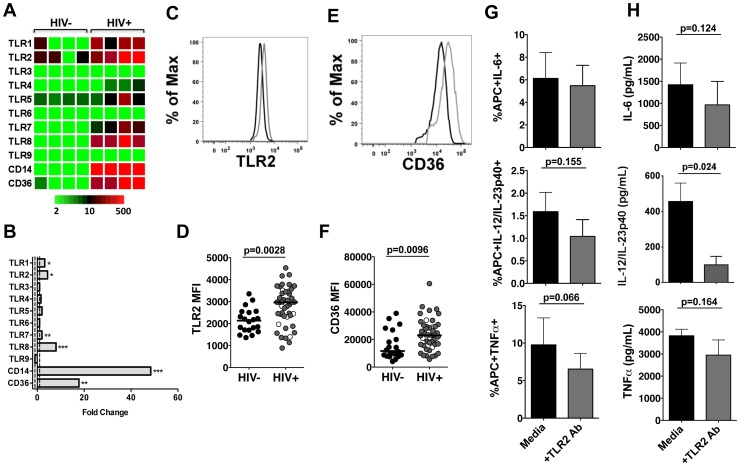
Increased expression of pattern recognition receptors on APCs from HIV-infected patients. (**A**) Heatmap and (**B**) fold changes of PRR gene expression in isolated CD11c+ APCs from HIV-infected patients were compared to HIV-negative controls (HIV− n = 4, HIV+ n = 4) using DNA microarray analysis. P values determined by unpaired t test (*P<0.05, **P<0.01, ***P<0.001). (**C**) Representative flow cytometry histograms of TLR2 expression of APCs from HIV-negative controls (black) and HIV+ patients (gray). (**D**) Median fluorescence intensity of TLR2 expression of APCs from HIV-negative controls (n = 20) and HIV-infected patients (n = 46). (**E**) Representative flow cytometry histograms of CD36 expression of APCs from HIV-negative controls (black) and HIV+ patients (gray). (**F**) Median fluorescence intensity of CD36 expression of APCs from HIV-negative controls (n = 20) and HIV-infected patients (n = 46). (**G**) Frequencies of APCs from HIV-infected patients (n = 7) producing IL-6, IL-12/IL23p40, and TNFα in response to *L. plantarum* WCFS1 with or without TLR2 blocking antibody. (**H**) Concentrations of IL-6, IL-12/IL-23p40, and TNFα following stimulation with *L. plantarum* WCFS1 with or without TLR2 blocking antibody as determined by ELISA (HIV+ n = 3–6). Each dot represents an individual subject. In the HIV+ group, open circles represent therapy-naïve patients, closed circles represent patients on HAART. Bars indicate median value. Bar graphs represent mean +/− SEM. P values determined using Mann Whitney U test or paired t test.

Recent studies demonstrate increased expression of pattern recognition receptors (PRRs), including TLR4, TLR2 and TLR2 co-receptor CD36, in both therapy-naïve and HAART-treated HIV-infected patients [10], [25]. Therefore, it is likely that increases in PRRs contribute to the observed APC hyper-responsiveness to commensal lactobacilli in HIV-infected patients. To investigate the impact of chronic, untreated HIV infection on peripheral blood APCs, we evaluated global gene expression profiles of APCs using DNA microarray analysis. CD11c+ APCs were isolated from four HIV-negative controls and four chronically infected, therapy-naïve patients with depleted CD4+ T-cells (84–637 cells/mm^3^) and plasma HIV RNA viral loads ranging from 15,485–976,464 HIV RNA copies/mL. DNA microarray analysis revealed distinct changes in APC gene expression profiles of pattern recognition receptors, specifically TLRs, between the HIV-infected patients and HIV-negative controls (Figure 3A). HIV infection was associated with significantly increased gene expression levels of TLRs that recognize bacterial ligands, including TLR1 and TLR2, as well as those that recognize viral RNA, including TLR7 and TLR8 (Figure 3B). Substantial increases in expression of co-receptors CD14 and CD36 transcripts were also found in HIV-infected patients (Figure 3B).

We focused further examination on TLR2 and its co-receptor CD36 because these PRRs play an important role in recognition of Gram-positive lactobacilli. We performed multicolor flow cytometry to determine whether cell surface expression of TLR2 and CD36 was increased in HIV infection. The median fluorescence intensity (MFI) of TLR2 on APCs was significantly higher in HIV-infected patients (Figure 3C, D). Increased MFI of CD36 was also detected on APCs from HIV-infected patients (Figure 3E, F).

To further investigate the role of increased TLR2 and CD36 expression in the enhanced APC inflammatory response to *L. plantarum* WCFS1, we performed a blocking assay. Because CD36 functions as a co-receptor that shuttles the ligand to TLR2, we chose to block TLR2 activation using an anti-TLR2 antibody [26], [27]. Blocking TLR2 reduced the frequencies of APCs producing proinflammatory cytokines (Figure 3G) as well as decreased the concentrations of IL-6, IL-12/IL23p40, and TNFα produced by PBMCs in response to *L. plantarum* WCFS1 (Figure 3H). Production of IL-12/IL-23p40 by PBMCs was significantly reduced with the TLR2 blocking treatment; however TLR2 blocking did not result in statistically significant reductions in PBMC production of IL-6 or TNFα following stimulation with *L. plantarum* WCFS1 (Figure 3H). These data indicate that increased TLR2 and CD36 expression contribute to the enhanced APC inflammatory response, but additional mechanisms might also contribute to the enhanced APC inflammatory response to commensal lactobacilli observed in HIV infection likely exist.

### p38-MAPK Signaling Drives the Heightened APC Inflammatory Response in HIV Infection

**Figure 4:**
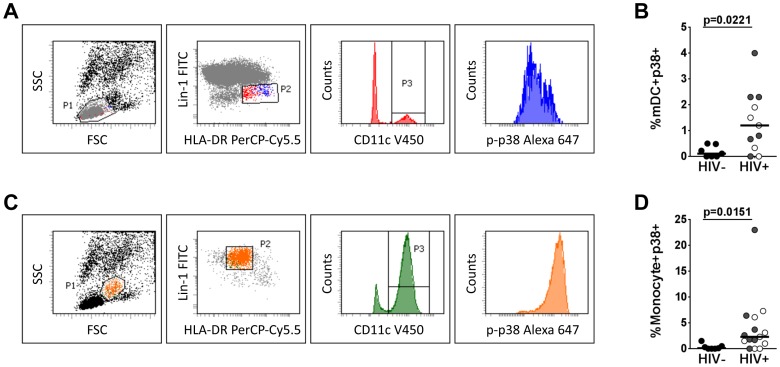
Increased phosphorylation of p38-MAPK in mDCs and monocytes from HIV-infected patients following stimulation with *L. plantarum*. (**A**) Representative flow cytometric gating strategy detecting phosphorylation of p38 in mDCs following stimulation with *L. plantarum* WCFS1. Myeloid dendritic cells were gated based on forward and side scatter followed by lineage-negative (CD3-CD14-CD16-CD19-CD20-CD56-), and were positive for both HLA-DR and CD11c. (**B**) Frequencies of mDCs phosphorylated p38 following stimulation with *L. plantarum* WCFS1 as determined by phosflow (HIV− n = 7; HIV+ n = 14). (**C**) Representative flow cytometric gating strategy detecting phosphorylation of p38 in monocytes following stimulation with *L. plantarum* WCFS1. Monocytes were gated based on forward and side scatter and were positive for the lineage marker, HLA-DR, and CD11c. (**D**) Frequencies of monocytes phosphorylated p38 following stimulation with *L. plantarum* WCFS1 as determined by phosflow (HIV− n = 7; HIV+ n = 14). Each dot represents an individual subject and bars indicate median. P values determined using Mann Whitney U test.

**Figure 5:**
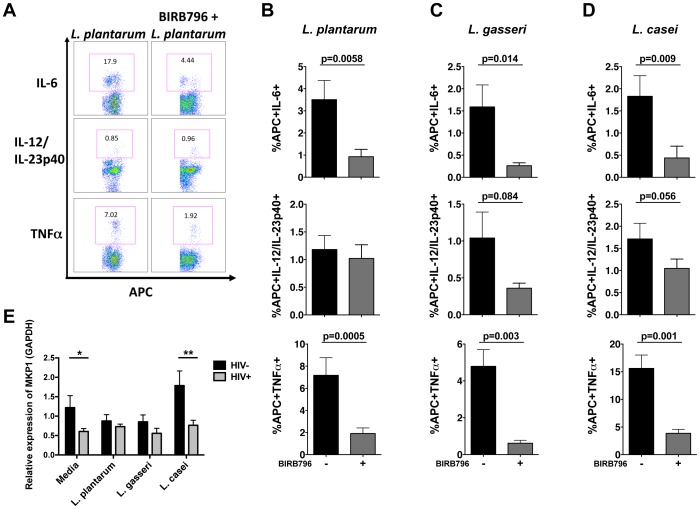
Enhanced APC inflammatory response to commensal lactobacilli signals predominantly through p38-MAPK. (**A**) Representative flow cytometry plot and frequencies of APCs from HIV-infected patients producing proinflammatory cytokines IL-6, IL-12/IL-23p40, and TNFα in response to (**B**) *L. plantarum* WCFS1 (n = 16), (**C**) *L. gasseri* 1SL4 (n = 12), and (**D**) *L. casei* BL23 (n = 11) with or without BIRB796 pretreatment. (**E**) Relative expression of MKP-1 in unstimulated and bacterial stimulated APCs as determined by real-time PCR (HIV− n = 3–9, HIV+ n = 5–16). Bar graphs represent mean +/− SEM. P values determined using paired t or Mann Whitney U test (*P<0.05, **P<0.01, ***P<0.001).

NFkB and p38-MAPK signaling pathways are important for producing inflammatory cytokines in response to bacteria [11], [28], [29]. Therefore, we measured NFkB and p38 phosphorylation in APC subsets following *L. plantarum* WCFS1 stimulation. While no significant difference in NFkB phosphorylation was found in APCs between the patient groups (Figure S3), higher frequencies of both mDCs (Figure 4A, B) and monocytes (Figure 4C, D) exhibited p38 phosphorylation following stimulation with *L. plantarum* WCFS1 in HIV-infected patients. To further validate the role of the p38-MAPK pathway in the enhanced APC response to *Lactobacillus* species, APCs from HIV-infected patients were pretreated with the p38-inhibitor, BIRB796, and exposed to commensal lactobacilli. Blocking p38 phosphorylation significantly reduced the frequencies of APCs producing IL-6 and TNFα in response to *L. plantarum* WCFS1 (Figure 5A and B). Significant reductions in the frequencies of APCs producing IL-6, IL-12/IL23p40, and TNFα in response to *L. gasseri* 1SL4 (Figure 5C) and *L. casei* BL23 (Figure 5D), as well as *L. rhamnosus* GG and *L. reuteri* F275 (Figure S2C, S2D), were also observed following BIRB796 treatment of PBMCs from HIV-infected patients. These data suggest that the p38-MAPK pathway plays an important role in the aberrant APC response to lactobacilli in HIV infection.

MAP kinase phosphatase-1 (MKP-1) regulates p38 phosphorylation and controls inflammation [30], [31]. Since our data demonstrated increased APC inflammatory cytokine production by APCs and increased p38 phosphorylation in HIV infection, we sought to determine whether HIV infection altered MKP-1 expression. APCs from HIV-infected patients demonstrated significantly reduced levels of MKP-1 (Figure 5E). In addition, a marked induction of MKP-1 mRNA levels in response to *L. casei* BL23 stimulation was observed only in APCs from HIV-negative controls (Figure 5E). These findings support the role of p38-MAPK signaling in HIV-induced alteration in inflammatory response of APCs to commensal lactobacilli.

## Discussion

The appropriate response of immune cells, particularly APCs, to both pathogenic and commensal bacteria is critical for human health, but may be especially complex in individuals with HIV infection. Previous HIV investigations have focused on identifying impaired immune cell responses to HIV as well as to pathogenic co-infections. In contrast, less is known about how HIV affects the immune response to commensal bacteria. In the present study, we examined the effects of HIV infection on APC response to commensal lactobacilli. APCs play a pivotal role in the host immune response as they control infections by generating an inflammatory response to pathogens but also maintain a tolerance to self and commensal bacteria so that excessive inflammation and tissue damage is avoided. Understanding the interaction of APCs with commensal bacteria in the setting of HIV infection is important since humans typically harbor over 10^14^ bacteria. We found that peripheral blood APCs from HIV-infected patients were hyper-responsive to stimulation with commensal lactobacilli. As expected, APCs from healthy, HIV-negative controls were generally non-responsive to stimulation with the lactobacilli that have previously been shown to induce minimal inflammatory response. However, APCs from HIV-infected patients produced higher levels of inflammatory cytokines in response to the lactobacilli stimulation. Collectively, these results provide evidence that HIV infection may induce enhanced APC inflammatory response to commensal lactobacilli.

Our data show heightened inflammatory response to commensal lactobacilli in both therapy-naïve and HAART-treated subject groups. Reflective of varying degrees of immune control of the virus as well as different magnitudes of CD4+ T-cell depletion, the therapy-naïve patients included in this study had a range of viral loads (<50–976,464 copies/mL plasma) and T-cells numbers (18–1353 cells/mm^3^). Four therapy-naïve patients had >700 CD4+ T-cells/mm^3^ and undetectable plasma viral loads despite being HIV-positive for several years. These patients likely have slower disease progression and maintain better APC functionality. Similarly, HAART-treated patients had varying levels of immune restoration, shown through the range of CD4+ T-cells (96–1578 cells per mm^3^). Seventeen HAART-treated patients had peripheral CD4+ T-cells counts <300 cells/mm^3^. In support of other studies, our data show that despite viral suppression in HAART-treatment, significant changes in immune cell function persist [3], [32]. In addition, increased immune activation and aberrant APC responses to lactobacilli were detected in a majority of HIV-infected patients.

Our findings support increased expression of TLR2 and CD36 in APCs from HIV-infected patients [10], [25], [33]. Increased expression of PRRs in HIV infection may lower the activation threshold of APCs, thereby enhancing their inflammatory response and permitting an inflammatory response to otherwise innocuous stimuli such as commensal lactobacilli. We focused our investigations on TLR2 and CD36, however blocking TLR2 using an anti-TLR2 antibody decreased the frequencies but did not completely abolish of APCs producing inflammatory cytokines in HIV-infected patients. This suggests that chronic HIV infection and the accompanying persistent immune activation upregulates additional PRRs (both known and unknown), which may subsequently contribute to the increased inflammatory response to lactobacilli observed in HIV-infected patients. It is also possible that HIV infection downregulates receptors that regulate or inhibit the inflammatory response which would also contribute to the observed APC response to lactobacilli. In addition, increased TLR expression and enhanced inflammatory response to intestinal microbiota have been reported in several chronic viral infections and chronic inflammatory conditions, including hepatitis C, CMV, and Crohn’s disease [2], [34], [35]. Since viruses, including HIV, activate TLRs 3, 7, 8 and 9, it is possible that immune cell responses to bacterial stimuli are altered as a consequence of TLR activation by the virus. Our study suggests that chronic viral infections or chronic inflammatory conditions may alter the APC expression of PRRs, thereby enhancing their response to commensal lactobacilli. Additional research is needed to determine the factor or combination of factors that cause increased TLR expression and the subsequent enhanced inflammatory response by APCs in HIV infection.

The p38-MAPK pathway is activated via TLR activation and results in the production of inflammatory cytokines. Increased expression of TLR2 likely contributes to the increased p38-MAPK phosphorylation in APC subsets from HIV-infected patients. It is also likely that increases in p38 phosphorylation may be influenced by the decreased expression of p38 regulator, MKP-1, in HIV-infected patients. Deficiencies in MKP-1 lead to excessive inflammation and play a role in desensitization to TLR activation [36], [37]. To our knowledge, we are the first to report decreased expression of MKP-1 in both unstimulated and bacterial-stimulated APCs from HIV-infected patients. Diminished basal MKP-1 expression, as well as expression following bacterial stimulation, in APCs from HIV-infected patients may contribute to increases in p38 phosphorylation and enhanced inflammatory response to commensal lactobacilli bacteria in HIV-infected patients. However, because blocking p38 did not completely abrogate the production of inflammatory cytokines by APCs, signaling pathways in addition to the p38 MAPK pathway are potentially involved in the aberrant APC response to lactobacilli species.

Based on our findings, we provide evidence that chronic HIV infection is associated with altered APC recognition and inflammatory response to commensal lactobacilli. We believe that chronic HIV infection leads to upregulation of TLR2 and CD36 expression. This upregulation may magnify APC sensitivity to bacteria subsequently leading to a more robust inflammatory response that signals, at least in part, through p38-MAPK. The resultant induction of proinflammatory cytokines by APCs in the event of translocation of small numbers of *Lactobacillus* or *Lactobacillus*-secreted metabolites across the GI lumen, could contribute to the higher plasma concentration of IL-6 and TNFα observed in HIV-infected patients.

Although our study focused on the aberrant inflammatory response of APCs from HIV-infected patients, it is probable that other immune cells contribute either directly or indirectly to the heightened inflammatory response to commensal lactobacilli in HIV-infected patients. Since immune cells communicate and work together and do not entirely function in isolation, we performed functional assays utilizing PBMCs rather than isolated APCs in order to adequately reflect a physiological response. While APCs are major producers of inflammatory cytokines in response to bacteria, we cannot rule out the possibility that additional immune cells were contributing to the enhanced inflammatory cytokine response. It is well known that HIV infection alters immune cell populations, particularly T cell subsets, as well as their functional responses. Therefore, it is likely that alterations or defects in T cell subsets, including regulatory T cells and Th17 cells, play an important role in possibly suppressing the enhanced APC inflammatory response in HIV-negative controls or secreting factors that induce the enhanced inflammatory response in HIV-infected patients. Additional research is needed to completely unravel the mechanisms contributing to the enhanced inflammatory response by APCs in HIV-infected patients and the contribution of other immune cells.

The effect of pathogens and chronic infections on the synergistic and beneficial relationship between the host immune system and commensal bacteria remains an under-investigated area of research. Our findings and previously reported studies suggest that the presence of one pathogen can alter host response to subsequent commensal microbial encounters [34], [38], [39]. In this regard, further investigations of chronic viral infections may identify common mechanisms that contribute to persistent immune activation through dysfunctional APC response to commensal bacteria. These findings are of high clinical significance since several *Lactobacillus* strains are used as probiotics and are becoming increasingly popular health supplements used to regulate digestion or to alleviate complications of inflammatory bowel diseases. However, the use of probiotics in HIV clinical trials to prevent and/or restore epithelial barrier disruption and subsequently decrease disease co-morbidities has produced conflicting results with some studies reporting benefits whereas others suggest the probiotics provide no clinical benefit [14], [15], [40], [41]. While the results and conclusions of our data are limited to the *Lactobacillus* strains examined, our findings highlight the importance of investigating APC inflammatory response to commensal bacteria. Further evaluation of APC responses to additional commensal bacterial species, both Gram-positive and Gram-negative, will shed light on those potential probiotic strains and/or therapeutics that truly dampen inflammation, thus preventing gut epithelial barrier disruption and HIV co-morbidities. Since many different bacterial species, and even some yeasts species, are employed as probiotics, it is possible that some of the strains are more beneficial than others. Therefore, it is important to evaluate the individual bacterial strains and determine the mechanisms by which they exert their beneficial effects to produce more targeted therapeutics for individuals with chronic viral infections such as HIV.

## Materials and Methods

### Subject Cohorts and Sample Collection

Peripheral blood samples were obtained from human study participants in three groups: HIV-1 infected therapy naïve, HIV-1 infected on long-term HAART, and HIV-negative controls. The inclusion criteria for the HIV-infected therapy-naïve group included positive diagnosis with HIV infection for at least one year and no prior history of anti-retroviral therapy (-Tx, n = 34). HIV-infected therapy-naïve subjects had peripheral blood CD4+ T cell counts of 18 to 1353 cells/mm^3^ and plasma viral loads ranging from <50 to 976,464 copies/mL. Four of 34 subjects in this group were HIV-positive for over 10 years but had plasma viral loads <50 copies/mL and maintained CD4+ T cell counts >700 cells/mm^3^. These subjects meet the criteria of long-term non-progressors. Three of the 34 subjects in the group also maintained high CD4+ T cell numbers in peripheral blood and plasma viral loads <5000 copies/mL. These subjects were known to be HIV-positive for less than five years and were likely in the clinically asymptomatic phase of infection. The remaining 27 HIV-infected therapy naïve subjects had variable degrees of CD4+ T cell loss and plasma viral loads.

Subjects included in the HIV-infected HAART-treated group were receiving HAART for at least 2 years and had undetectable plasma viral loads (<50 HIV RNA copies/mL) (+Tx, n = 63). The CD4+ T cell numbers in peripheral blood samples ranged from 96 to 1578 cells/mm^3^ suggesting variable level of CD4+ T cell recovery in response to therapy. Seventeen of the 60 HAART-treated subjects had peripheral blood CD4+ T cells counts below 300 cells/mm^3^.

Healthy subjects in the HIV-negative control group included those with no prior diagnosis of HIV infection or chronic disease (HIV−, n = 40). Due to variability in cell yields from the peripheral blood samples from the HIV-infected patients, each assay was not performed on every patient sample. Each figure contains the sample size (n) for each assay. Studies were performed under informed written consent and a protocol approved by the University of California-Davis Institutional Review Board.

### Peripheral Blood Mononuclear Cell Isolation

Whole blood was collected using EDTA tubes. PBMCs were isolated by methods previously described [42]. Following isolation, cells were washed in 1X PBS (GIBCO), pelleted, and resuspended in media with no antibiotics (RPMI-1640 supplemented with 10% FBS, 1% L-glutamine, and 1% HEPES, GIBCO).

### Immunophenotypic Analysis

Multicolor flow cytometry was used for phenotypic analysis. Samples were stained with antibodies for surface markers CD3 (SP34-2), CD4 (OKT4), CD8 (RPA-T8), CD19 (H1B19), CD56 (HCD56), HLA-DR (L243), CD11c (3.9), CD123 (7G3), CD14 (TUK4), TLR2 (TL2.1), and CD36 (5-271) (BD Bioscience, Invitrogen, Biolegend, eBioscience). Cells were washed, fixed in 1% PFA, and analyzed with LSRII (BD Bioscience). Data analysis was performed using FlowJo software (TreeStar).

The APC gating strategy is as follows: PBMCs were initially gated based on forward and side scatter to eliminate debris and non-immune cells. Exclusion of doublets was included to ensure analysis of the APC population was based on single cells. Cell viability was assessed using LIVE/DEAD Fixable Dead Cell Stain kit (Invitrogen) and dead cells were excluded. Cells expressing CD3 (T cells), CD19 (B cells), and CD56 (NK cells) were excluded. Cells positive for HLA-DR were gated for CD11c and CD123. APCs were defined as CD11c-positive and CD123-negative. From the APC population, the monocyte and mDC populations were identified using the CD14 marker. Monocytes were defined as cells positive for CD14 expression and included cells expressing both low to high levels of CD14. Cells negative for CD14 but positive for CD11c expression were considered mDCs.

### Plasma sCD14 Detection

Plasma levels of sCD14 were determined using Quantikine ELISA (R & D Systems) as per manufacturer’s instructions.

### Bacterial Stimulation

PBMCs were exposed to *Lactobacillus plantarum* WCFS1 [19], *Lactobacillus gasseri* 1SL4 (ATCC 19992) [43], *Lactobacillus casei* BL23 [44], *Lactobacillus rhamnosus* GG (ATCC 53101) [45], and *Lactobacillus reuteri* F275 (ATCC 23272) [43], were grown in MRS broth (BD Biosciences) overnight, washed in PBS, and were incubated with PBMCs at a multiplicity of infection (MOI) of 1∶2. Samples were incubated at 37°C/5% CO_2_ for one hour then washed with 1X PBS. Cells were resuspended in media containing antibiotics (RPMI-1640, 10% FBS, 1% Penicillin/Streptomycin/Glutamine, and 1% HEPES), Brefeldin-A was added and cells were incubated for an additional three hours. Samples were washed and stained with antibodies for surface markers CD3, CD19, CD56, HLA-DR, CD11c, CD123, and CD14. Cells were washed and fixed, then permeabilized with Caltag Permeabilization Solution B (Invitrogen) and stained with antibodies for cytokines IL-12/IL-23p40 (C8.6), TNFα (MAb11), and IL-6 (MQ2-13A5) (eBioscience). Cells were washed, fixed, and analyzed with LSRII (BD Bioscience). Data analysis was performed using FlowJo software (TreeStar).

For ELISA assays, 2×10^6^ PBMCs from randomly selected HIV-negative (n = 4) and ten HIV-infected patients (therapy naïve n = 2, HAART n = 8) were resuspended in 500 µl of media with no antibiotics and stimulated with *L. plantarum* WCFS1 at an MOI of 1∶2.After one hour of stimulation, an additional 500 µl of media with antibiotics was added and the cells were incubated for three additional hours. Following stimulation, cell supernatants were collected and frozen at −80°C. Supernatants were thawed and IL-6, IL-12/23p40, and TNFα ELISAs (ELISA MAX Deluxe, Biolegend) were performed following manufacturer’s protocol.

### TLR2 Blocking Assay

PBMCs from HIV-infected patients were pretreated with 1 µg/mL anti-TLR2 antibody (TL2.1, eBioscience) then stimulated with *L. plantarum* WCFS1 as described above. For ELISAs, PBMCs from HIV-infected patients were pretreated with 1 µg/mL anti-TLR2 antibody (T2.5, Biolegend) for one hour then stimulated with *L. plantarum* WCFS1 for four hours in media with no antibiotics. Following stimulation, cell supernatants were collected and frozen at −80°C. Supernatants were thawed and IL-6, IL-12/23p40, and TNFα ELISAs (ELISA MAX Deluxe, Biolegend) were run following manufacturer’s protocol.

### Phospho-protein Detection

Phospho-proteins analysis by flow cytometry was performed following the protocol as previously described [46]. PBMCs were harvested by centrifugation using CPT tubes (BD Biosciences). Cells were washed, resuspended in PBS and stained for 20 minutes at room temperature with Lin-1 FITC and CD11c V450 (BD Biosciences). Cells were then stimulated with *L. plantarum* WCFS1 at an MOI of 1∶10 for 20 minutes at 37°C. Following stimulation, cells were fixed, washed, permeabilized using BD Custom perm buffer (BD Biosciences) and stained for one hour at room temperature with anti-NFkB (pS529) PE, anti-p38 MAPK (pT180/pY182) Alexa 647 and HLA-DR PerCP-Cy5.5 (BD Biosciences). After a final wash, the cells were analyzed on BD FACSCanto™ II flow cytometer.

### p38-MAPK Inhibition

Following two hours of incubation with 10 mM BIRB796 (Axon Medchem), bacterial stimulations were performed as previously described.

### MKP-1 Expression Analysis

PBMCs were enriched for APCs using magnetic Pan Mouse IgG beads (Dynabeads, Invitrogen) conjugated to CD3, CD19, CD56, and CD11c antibodies. After CD3^+^, CD19^+^, and CD56^+^ cells were removed from PBMCs, CD11c conjugated beads were used to positively enrich the APC population. APCs were stimulated with bacteria (MOI of 1∶2). Samples were incubated at 37°C/5% CO_2_ for one hour, pelleted and snap-frozen for real-time PCR. RNA was extracted using the RNeasy RNA isolation kit (Qiagen). Isolated RNA was treated with DNase (Invitrogen) and reverse transcribed using the superscript III first strand synthesis kit (Invitrogen). Real time PCR was performed using 2x Taqman universal master mix (Applied Biosystems) and a specific MKP1 primer (predesigned assay Hs00610256_g1; Applied Biosystems) on a Viia 7 real-time PCR system. Gene expression was normalized to GAPDH levels (Applied Biosystems).

### DNA Microarray Analysis

CD11c^+^ APCs were isolated from PBMCs from four HIV-negative controls and four therapy-naïve subjects with high levels of plasma viral loads (15,485-976,464 HIV RNA copies/mL plasma) and CD4+ T cells from 84-637 cells/mm^3^. Dynabeads CD8 and Dynabeads Pan Mouse IgG (Invitrogen) beads conjugated to CD3, CD13, or CD11c antibodies were utilized to enrich the APC population. Following selection of CD3^+^, CD8^+^, and CD13^+^ cells, the remaining PBMCs were positively selected for CD11c^+^ APCs and snap frozen. Total RNA was extracted from isolated APCs utilizing Qiagen RNeasy RNA isolation kit (Qiagen). mRNA amplification, labeling, hybridization to human genome GeneChips (Affymetrix), staining, and scanning were performed according to the Affymetrix Gene Expression Analysis Technical Manual at the Microarray Core Facility at the University of California-Davis [47], [48]. Stringent statistical criteria were applied to the microarray data analysis using RMA-based (GeneSpring, Agilent Technologies) algorithms. A minimum fold-change of 50% (*p-*value ≤0.05) was used as a cut off. Genes meeting fold-change and statistical criteria were functionally categorized and pathways and processes statistically enriched were identified with dChip and Ingenuity Pathway Analysis software. The microarray data set is deposited at the Gene Expression Omnibus at the National Center for Biotechnology Information (GSE42058).

### Statistical Analysis

Statistical significance was assessed using Mann Whitney U test and paired t tests were used to determine statistical significance as indicated. Statistics were generated using GraphPad Prism (GraphPad Software).

## Supporting Information

Figure S1 **Monocyte and myeloid dendritic cell inflammatory response to lactobacilli.** (**A**) Frequencies of monocytes producing IL-6, IL-12/IL-23p40, and TNFα in response to *L. plantarum* WCFS1 (HIV− n = 23, HIV+ n = 43), *L. gasseri* 1SL4 (HIV− n = 7, HIV+ n = 23), and *L. casei* BL23 (HIV− n = 7, HIV+ n = 23) as measured by multicolor flow cytometry. (**B**) Frequencies of mDCs producing IL-6, IL-12/IL-23p40, and TNFα in response to *L. plantarum* WCFS1 (HIV− n = 23, HIV+ n = 43), *L. gasseri* 1SL4 (HIV− n = 7, HIV+ n = 23), and *L. casei* BL23 (HIV− n = 7, HIV+ n = 23) as measured by multicolor flow cytometry. Each dot represents an individual subject. In the HIV+ group, open circles represent therapy-naïve patients, closed circles represent patients on HAART. Bars indicate median value. P values determined using Mann Whitney U test, P values as indicated.
(TIF)

Figure S2 **Enhanced inflammatory response by APCs from HIV-infected patients to commensal** ***L. rhamnosus*** **GG and** ***L. reuteri*** **F275 dampened by blocking p38-MAPK.** Frequencies of APCs from HIV-negative controls (n = 4) and HIV-infected patients (n = 7) producing IL-6, IL-12/IL-23p40, and TNFα in response to (A) *L. rhamnosus* GG and (B) *L. reuteri* F275 determined by multicolor flow cytometry. Each dot represents an individual subject. In the HIV+ group, open circles represent therapy-naïve patients, closed circles represent patients on HAART. Bars indicate median value. Bar graphs represent mean +/− SEM. P values determined using Mann Whitney U test. Frequencies of APCs from HIV-infected patients (n = 4) producing IL-6, IL-12/IL-23p40, and TNFα in response to (C) *L. rhamnosus* GG and (D) *L. reuteri* F275 with or without BIRB796 pretreatment. Bar graphs represent mean +/− SEM. P values determined using paired t test.
(TIF)

Figure S3 **Similar phosphorylation of NFkB in HIV-infected patients and HIV-negative controls following stimulation with** ***L. plantarum*** **WCFS1.** Frequencies of monocytes and mDCs with phosphorylated NFkB following stimulation with *L. plantarum* WCFS1 as determined by phosflow (HIV− n = 7; HIV+ n = 11). Each dot represents an individual subject and bars indicate median. In the HIV+ group, open circles represent therapy-naïve patients and closed, gray circles represent those on HAART. P values determined using Mann Whitney U test.
(TIF)

## References

[1] Liu Z, Cumberland WG, Hultin LE, Prince HE, Detels R, et al (1997) Elevated CD38 antigen expression on CD8+ T cells is a stronger marker for the risk of chronic HIV disease progression to AIDS and death in the Multicenter AIDS Cohort Study than CD4+ cell count, soluble immune activation markers, or combinations of HLA-DR and CD38 expression. J Acquir Immune Defic Syndr Hum Retrovirol 16: 83–92. 9358102 10.1097/00042560-199710010-00003

[2] Roberts L, Passmore JA, Williamson C, Little F, Bebell LM, et al (2010) Plasma cytokine levels during acute HIV-1 infection predict HIV disease progression. AIDS 24: 819–831. 20224308 10.1097/QAD.0b013e3283367836 PMC3001189

[3] Price P, Mathiot N, Krueger R, Stone S, Keane NM, et al (2001) Immune dysfunction and immune restoration disease in HIV patients given highly active antiretroviral therapy. J Clin Virol 22: 279–287. 11564593 10.1016/s1386-6532(01)00200-1

[4] Guadalupe M, Sankaran S, George MD, Reay E, Verhoeven D, et al (2006) Viral suppression and immune restoration in the gastrointestinal mucosa of human immunodeficiency virus type 1-infected patients initiating therapy during primary or chronic infection. J Virol 80: 8236–8247. 16873279 10.1128/JVI.00120-06 PMC1563811

[5] Sharkey ME, Teo I, Greenough T, Sharova N, Luzuriaga K, et al (2000) Persistence of episomal HIV-1 infection intermediates in patients on highly active anti-retroviral therapy. Nat Med 6: 76–81. 10613828 10.1038/71569 PMC9513718

[6] Brenchley JM, Price DA, Schacker TW, Asher TE, Silvestri G, et al (2006) Microbial translocation is a cause of systemic immune activation in chronic HIV infection. Nat Med 12: 1365–1371. 17115046 10.1038/nm1511

[7] Medzhitov R (2001) Toll-like receptors and innate immunity. Nat Rev Immunol 1: 135–145. 11905821 10.1038/35100529

[8] Akira S, Takeda K (2004) Toll-like receptor signalling. Nat Rev Immunol 4: 499–511. 15229469 10.1038/nri1391

[9] Eckburg PB, Bik EM, Bernstein CN, Purdom E, Dethlefsen L, et al (2005) Diversity of the human intestinal microbial flora. Science 308: 1635–1638. 15831718 10.1126/science.1110591 PMC1395357

[10] Meroni L, Riva A, Morelli P, Galazzi M, Mologni D, et al (2005) Increased CD36 expression on circulating monocytes during HIV infection. J Acquir Immune Defic Syndr 38: 310–313. 15735450

[11] Xiao J, Qian KL, Cao QH, Qiu CL, Qiu C, et al (2011) HLA-DR expression on regulatory T cells is closely associated with the global immune activation in HIV-1 infected subjects naive to antiretroviral therapy. Chin Med J (Engl) 124: 2340–2346. 21933566

[12] Jiang W, Lederman MM, Hunt P, Sieg SF, Haley K, et al (2009) Plasma levels of bacterial DNA correlate with immune activation and the magnitude of immune restoration in persons with antiretroviral-treated HIV infection. J Infect Dis 199: 1177–1185. 19265479 10.1086/597476 PMC2728622

[13] Merlini E, Bai F, Bellistri GM, Tincati C, d’Arminio Monforte A, et al (2011) Evidence for polymicrobic flora translocating in peripheral blood of HIV-infected patients with poor immune response to antiretroviral therapy. PLoS One 6: e18580. 21494598 10.1371/journal.pone.0018580 PMC3073938

[14] Cunningham-Rundles S, Ahrne S, Johann-Liang R, Abuav R, Dunn-Navarra AM, et al (2011) Effect of probiotic bacteria on microbial host defense, growth, and immune function in human immunodeficiency virus type-1 infection. Nutrients 3: 1042–1070. 22292110 10.3390/nu3121042 PMC3260491

[15] Anukam KC, Osazuwa EO, Osadolor HB, Bruce AW, Reid G (2008) Yogurt containing probiotic Lactobacillus rhamnosus GR-1 and L. reuteri RC-14 helps resolve moderate diarrhea and increases CD4 count in HIV/AIDS patients. J Clin Gastroenterol 42: 239–243. 18223503 10.1097/MCG.0b013e31802c7465

[16] Ancuta P, Kamat A, Kunstman KJ, Kim EY, Autissier P, et al (2008) Microbial translocation is associated with increased monocyte activation and dementia in AIDS patients. PLoS One 3: e2516. 18575590 10.1371/journal.pone.0002516 PMC2424175

[17] Mendez-Lagares G, Romero-Sanchez MC, Ruiz-Mateos E, Genebat M, Ferrando-Martinez S, et al.. (2013) Long-Term Suppressive Combined Antiretroviral Treatment Does Not Normalize the Serum Level of Soluble CD14. J Infect Dis. 10.1093/infdis/jit025 23322858

[18] van Hemert S, Meijerink M, Molenaar D, Bron PA, de Vos P, et al (2010) Identification of Lactobacillus plantarum genes modulating the cytokine response of human peripheral blood mononuclear cells. BMC Microbiol 10: 293. 21080958 10.1186/1471-2180-10-293 PMC3000848

[19] Lenkei R, Bratt G, Holmberg V, Muirhead K, Sandstrom E (1998) Indicators of T-cell activation: correlation between quantitative CD38 expression and soluble CD8 levels in asymptomatic HIV+ individuals and healthy controls. Cytometry 33: 115–122. 9773871 10.1002/(sici)1097-0320(19981001)33:2<115::aid-cyto5>3.0.co;2-i

[20] Thomas CM, Versalovic J (2010) Probiotics-host communication: Modulation of signaling pathways in the intestine. Gut Microbes 1: 148–163. 20672012 10.4161/gmic.1.3.11712 PMC2909492

[21] Hemarajata P, Versalovic J (2013) Effects of probiotics on gut microbiota: mechanisms of intestinal immunomodulation and neuromodulation. Therap Adv Gastroenterol 6: 39–51. 10.1177/1756283X12459294 PMC3539293 23320049

[22] Jan RL, Yeh KC, Hsieh MH, Lin YL, Kao HF, et al (2012) Lactobacillus gasseri suppresses Th17 pro-inflammatory response and attenuates allergen-induced airway inflammation in a mouse model of allergic asthma. Br J Nutr 108: 130–139. 21996276 10.1017/S0007114511005265

[23] Rochat T, Bermudez-Humaran L, Gratadoux JJ, Fourage C, Hoebler C, et al (2007) Anti-inflammatory effects of Lactobacillus casei BL23 producing or not a manganese-dependant catalase on DSS-induced colitis in mice. Microb Cell Fact 6: 22. 17659075 10.1186/1475-2859-6-22 PMC1949835

[24] Watterlot L, Rochat T, Sokol H, Cherbuy C, Bouloufa I, et al (2010) Intragastric administration of a superoxide dismutase-producing recombinant Lactobacillus casei BL23 strain attenuates DSS colitis in mice. Int J Food Microbiol 144: 35–41. 20452077 10.1016/j.ijfoodmicro.2010.03.037

[25] Hernandez JC, Stevenson M, Latz E, Urcuqui-Inchima S (2012) HIV Type 1 Infection Up-Regulates TLR2 and TLR4 Expression and Function in Vivo and in Vitro. AIDS Res Hum Retroviruses. 10.1089/aid.2011.0297 PMC3482876 22280204

[26] Triantafilou M, Gamper FG, Haston RM, Mouratis MA, Morath S, et al (2006) Membrane sorting of toll-like receptor (TLR)-2/6 and TLR2/1 heterodimers at the cell surface determines heterotypic associations with CD36 and intracellular targeting. J Biol Chem 281: 31002–31011. 16880211 10.1074/jbc.M602794200

[27] Nilsen NJ, Deininger S, Nonstad U, Skjeldal F, Husebye H, et al (2008) Cellular trafficking of lipoteichoic acid and Toll-like receptor 2 in relation to signaling: role of CD14 and CD36. J Leukoc Biol 84: 280–291. 18458151 10.1189/jlb.0907656 PMC3178504

[28] van Baarlen P, Troost FJ, van Hemert S, van der Meer C, de Vos WM, et al (2009) Differential NF-kappaB pathways induction by Lactobacillus plantarum in the duodenum of healthy humans correlating with immune tolerance. Proc Natl Acad Sci U S A 106: 2371–2376. 19190178 10.1073/pnas.0809919106 PMC2650163

[29] Giahi L, Aumueller E, Elmadfa I, Haslberger AG (2012) Regulation of TLR4, p38 MAPkinase, IkappaB and miRNAs by inactivated strains of lactobacilli in human dendritic cells. Benef Microbes 3: 91–98. 22476320 10.3920/BM2011.0052

[30] Salojin KV, Owusu IB, Millerchip KA, Potter M, Platt KA, et al (2006) Essential role of MAPK phosphatase-1 in the negative control of innate immune responses. J Immunol 176: 1899–1907. 16424221 10.4049/jimmunol.176.3.1899

[31] Wang X, Meng X, Kuhlman JR, Nelin LD, Nicol KK, et al (2007) Knockout of Mkp-1 enhances the host inflammatory responses to gram-positive bacteria. J Immunol 178: 5312–5320. 17404316 10.4049/jimmunol.178.8.5312

[32] Fernandez S, Tanaskovic S, Helbig K, Rajasuriar R, Kramski M, et al (2011) CD4+ T-cell deficiency in HIV patients responding to antiretroviral therapy is associated with increased expression of interferon-stimulated genes in CD4+ T cells. J Infect Dis 204: 1927–1935. 22006994 10.1093/infdis/jir659

[33] Heggelund L, Muller F, Lien E, Yndestad A, Ueland T, et al (2004) Increased expression of toll-like receptor 2 on monocytes in HIV infection: possible roles in inflammation and viral replication. Clin Infect Dis 39: 264–269. 15307037 10.1086/421780

[34] Doisne JM, Urrutia A, Lacabaratz-Porret C, Goujard C, Meyer L, et al (2004) CD8+ T cells specific for EBV, cytomegalovirus, and influenza virus are activated during primary HIV infection. J Immunol 173: 2410–2418. 15294954 10.4049/jimmunol.173.4.2410

[35] Trial J, Birdsall HH, Hallum JA, Crane ML, Rodriguez-Barradas MC, et al (1995) Phenotypic and functional changes in peripheral blood monocytes during progression of human immunodeficiency virus infection. Effects of soluble immune complexes, cytokines, subcellular particulates from apoptotic cells, and HIV-1-encoded proteins on monocytes phagocytic function, oxidative burst, transendothelial migration, and cell surface phenotype. J Clin Invest 95: 1690–1701. 7706478 10.1172/JCI117845 PMC295681

[36] Frazier WJ, Wang X, Wancket LM, Li XA, Meng X, et al (2009) Increased inflammation, impaired bacterial clearance, and metabolic disruption after gram-negative sepsis in Mkp-1-deficient mice. J Immunol 183: 7411–7419. 19890037 10.4049/jimmunol.0804343 PMC2882055

[37] Wang J, Ford HR, Grishin AV (2010) NF-kappaB-mediated expression of MAPK phosphatase-1 is an early step in desensitization to TLR ligands in enterocytes. Mucosal Immunol 3: 523–534. 20555314 10.1038/mi.2010.35

[38] Hand TW, Dos Santos LM, Bouladoux N, Molloy MJ, Pagan AJ, et al.. (2012) Acute Gastrointestinal Infection Induces Long-Lived Microbiota-Specific T Cell Responses. Science. 10.1126/science.1220961 PMC3784339 22923434

[39] Haas A, Zimmermann K, Graw F, Slack E, Rusert P, et al (2011) Systemic antibody responses to gut commensal bacteria during chronic HIV-1 infection. Gut 60: 1506–1519. 21515549 10.1136/gut.2010.224774

[40] Klatt NR, Canary LA, Sun X, Vinton CL, Funderburg NT, et al.. (2013) Probiotic/prebiotic supplementation of antiretrovirals improves gastrointestinal immunity in SIV-infected macaques. J Clin Invest. 10.1172/JCI66227 PMC3561826 23321668

[41] Salminen MK, Tynkkynen S, Rautelin H, Poussa T, Saxelin M, et al (2004) The efficacy and safety of probiotic Lactobacillus rhamnosus GG on prolonged, noninfectious diarrhea in HIV Patients on antiretroviral therapy: a randomized, placebo-controlled, crossover study. HIV Clin Trials 5: 183–191. 15472792 10.1310/6F83-N39Q-9PPP-LMVV

[42] Guadalupe M, Reay E, Sankaran S, Prindiville T, Flamm J, et al (2003) Severe CD4+ T-cell depletion in gut lymphoid tissue during primary human immunodeficiency virus type 1 infection and substantial delay in restoration following highly active antiretroviral therapy. J Virol 77: 11708–11717. 14557656 10.1128/JVI.77.21.11708-11717.2003 PMC229357

[43] Mohamadzadeh M, Olson S, Kalina WV, Ruthel G, Demmin GL, et al (2005) Lactobacilli activate human dendritic cells that skew T cells toward T helper 1 polarization. Proc Natl Acad Sci U S A 102: 2880–2885. 15710900 10.1073/pnas.0500098102 PMC549474

[44] Maze A, Boel G, Zuniga M, Bourand A, Loux V, et al (2010) Complete genome sequence of the probiotic Lactobacillus casei strain BL23. J Bacteriol 192: 2647–2648. 20348264 10.1128/JB.00076-10 PMC2863562

[45] Kankainen M, Paulin L, Tynkkynen S, von Ossowski I, Reunanen J, et al (2009) Comparative genomic analysis of Lactobacillus rhamnosus GG reveals pili containing a human- mucus binding protein. Proc Natl Acad Sci U S A 106: 17193–17198. 19805152 10.1073/pnas.0908876106 PMC2746127

[46] Suni MA, Maino VC (2011) Flow cytometric analysis of cell signaling proteins. Methods Mol Biol 717: 155–169. 21370030 10.1007/978-1-61779-024-9_9

[47] Lerner P, Guadalupe M, Donovan R, Hung J, Flamm J, et al (2011) The gut mucosal viral reservoir in HIV-infected patients is not the major source of rebound plasma viremia following interruption of highly active antiretroviral therapy. J Virol 85: 4772–4782. 21345945 10.1128/JVI.02409-10 PMC3126205

[48] Sankaran S, George MD, Reay E, Guadalupe M, Flamm J, et al (2008) Rapid onset of intestinal epithelial barrier dysfunction in primary human immunodeficiency virus infection is driven by an imbalance between immune response and mucosal repair and regeneration. J Virol 82: 538–545. 17959677 10.1128/JVI.01449-07 PMC2224350

