# Supplementary figures and images for: Chronic HIV Infection Enhances the Responsiveness of Antigen Presenting Cells to Commensal Lactobacillus
Source: PLoS One. 2013 Aug 30;8(8):e72789. doi: 10.1371/journal.pone.0072789 (PMC3758347; doi:10.1371/journal.pone.0072789)

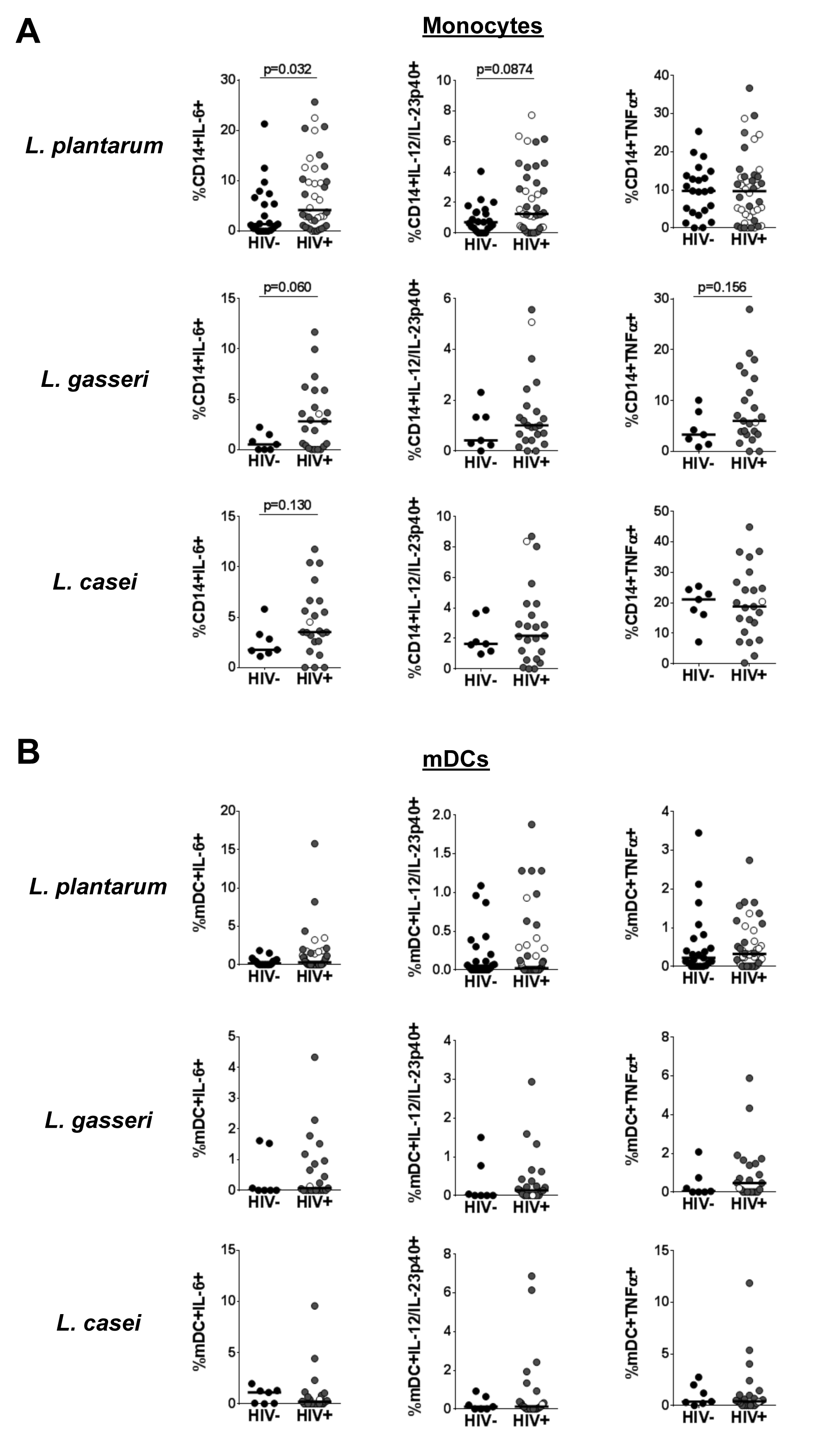

Supplement: Figure S1 — Monocyte and myeloid dendritic cell inflammatory response to lactobacilli. (A) Frequencies of monocytes producing IL-6, IL-12/IL-23p40, and TNFα in response to L. plantarum WCFS1 (HIV− n = 23, HIV+ n = 43), L. gasseri 1SL4 (HIV− n = 7, HIV+ n = 23), and L. casei BL23 (HIV− n = 7, HIV+ n = 23) as measured by multicolor flow cytometry. (B) Frequencies of mDCs producing IL-6, IL-12/IL-23p40, and TNFα in response to L. plantarum WCFS1 (HIV− n = 23, HIV+ n = 43), L. gasseri 1SL4 (HIV− n = 7, HIV+ n = 23), and L. casei BL23 (HIV− n = 7, HIV+ n = 23) as measured by multicolor flow cytometry. Each dot represents an individual subject. In the HIV+ group, open circles represent therapy-naïve patients, closed circles represent patients on HAART. Bars indicate median value. P values determined using Mann Whitney U test, P values as indicated. (TIF) [file pone.0072789.s001.tif]

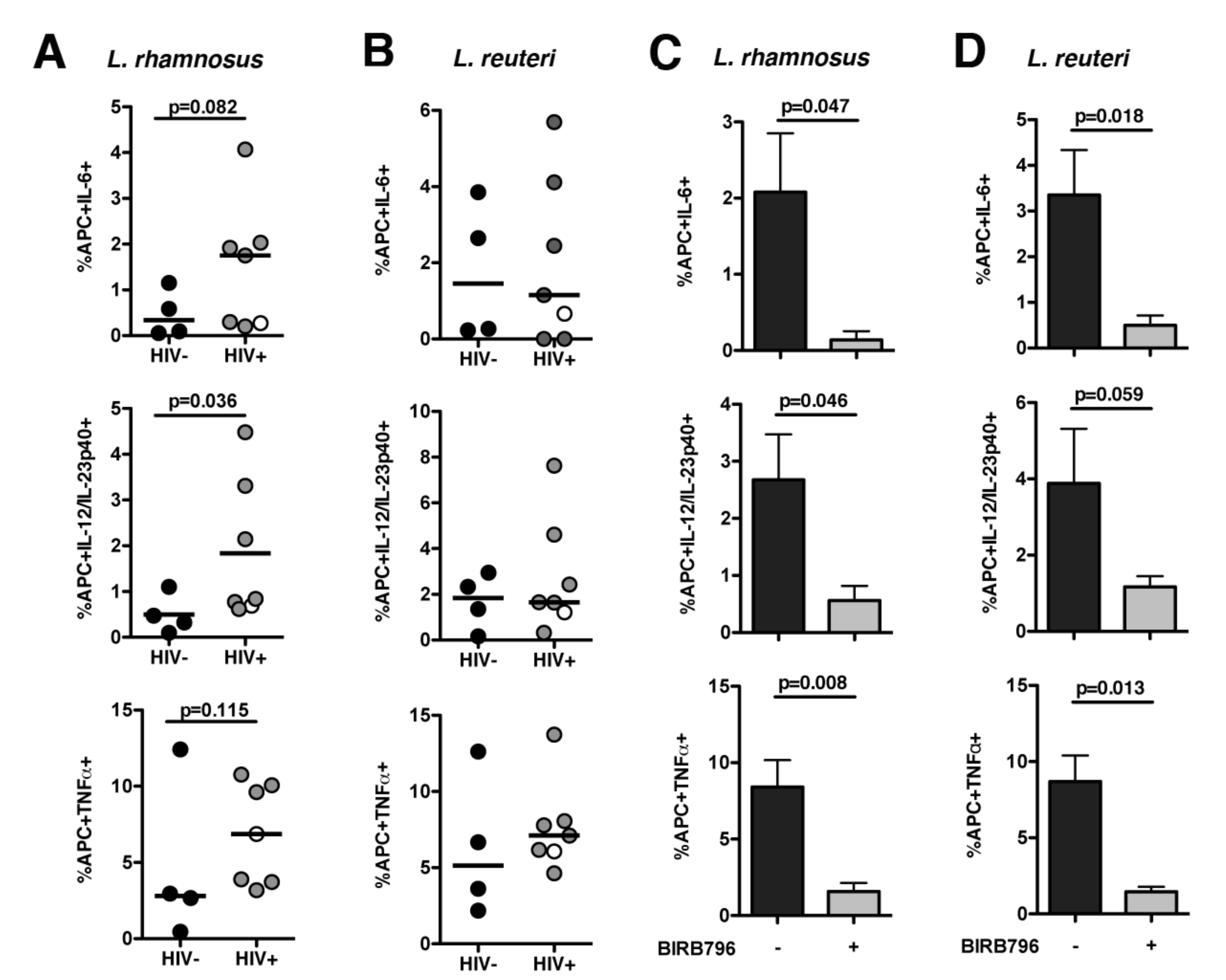

Supplement: Figure S2 — Enhanced inflammatory response by APCs from HIV-infected patients to commensal L. rhamnosus GG and L. reuteri F275 dampened by blocking p38-MAPK. Frequencies of APCs from HIV-negative controls (n = 4) and HIV-infected patients (n = 7) producing IL-6, IL-12/IL-23p40, and TNFα in response to (A) L. rhamnosus GG and (B) L. reuteri F275 determined by multicolor flow cytometry. Each dot represents an individual subject. In the HIV+ group, open circles represent therapy-naïve patients, closed circles represent patients on HAART. Bars indicate median value. Bar graphs represent mean +/− SEM. P values determined using Mann Whitney U test. Frequencies of APCs from HIV-infected patients (n = 4) producing IL-6, IL-12/IL-23p40, and TNFα in response to (C) L. rhamnosus GG and (D) L. reuteri F275 with or without BIRB796 pretreatment. Bar graphs represent mean +/− SEM. P values determined using paired t test. (TIF) [file pone.0072789.s002.tif]

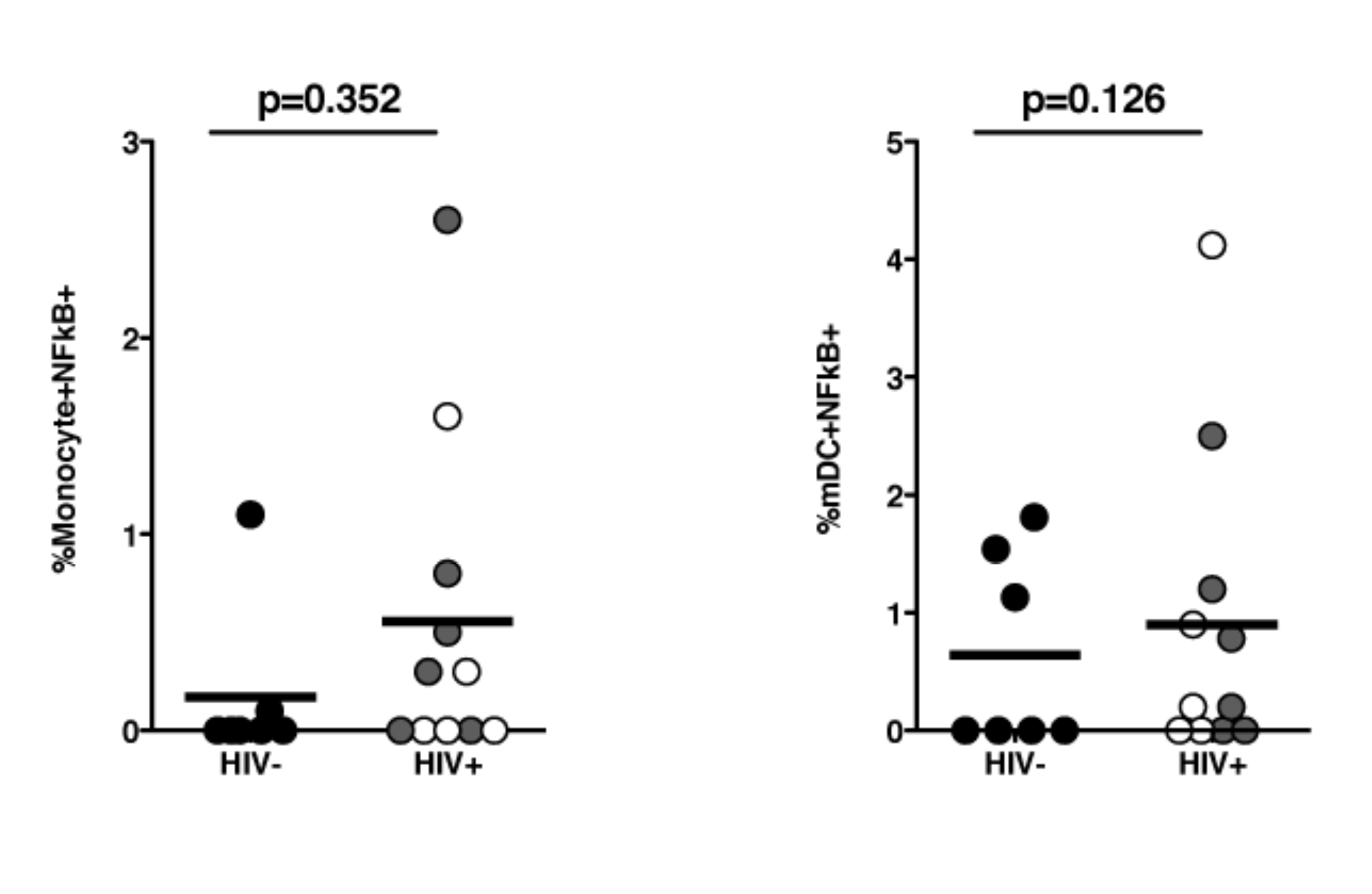

Supplement: Figure S3 — Similar phosphorylation of NFkB in HIV-infected patients and HIV-negative controls following stimulation with L. plantarum WCFS1. Frequencies of monocytes and mDCs with phosphorylated NFkB following stimulation with L. plantarum WCFS1 as determined by phosflow (HIV− n = 7; HIV+ n = 11). Each dot represents an individual subject and bars indicate median. In the HIV+ group, open circles represent therapy-naïve patients and closed, gray circles represent those on HAART. P values determined using Mann Whitney U test. (TIF) [file pone.0072789.s003.tif]
